# Malperfusion on Presentation Versus Complexity of Operation as Determinants of Early Mortality in Acute Type A Aortic Dissection

**DOI:** 10.1055/a-2833-4913

**Published:** 2026-03-26

**Authors:** Charles S. Roberts, Kyle A. McCullough, John B. Eisenga, J. Michael DiMaio

**Affiliations:** 1 Department of Cardiac Surgery, Baylor University Medical Center, Dallas, Texas, United States; 2 Department of Cardiovascular Research, Baylor Scott and White Research Institute, Plano, Texas, United States; 3 Department of Cardiac Surgery, Baylor Scott and White Heart Hospital, Plano, Texas, United States; 4 Department of Biomedical Engineering, Texas A&M University, College Station, Texas, United States

**Keywords:** aortic dissection, malperfusion, Penn Class, operative complexity

## Abstract

**Background:**

Degree of malperfusion on presentation is a known determinant of early mortality in acute Type A aortic dissection (TAAD). Its prediction of mortality when stratified by complexity of central repair has not been well-described.

**Methods:**

Over a 6-year period, 183 patients had a central repair for TAAD, 146 of whom had a spontaneous etiology and an acute presentation (≤14 days). Each patient was assigned a Penn Class based on ischemia (malperfusion): A-none, B-regional, or C-global. The index operation was identified as simple (ascending aorta and/or hemiarch replacement) or complex (concomitant root replacement, arch replacement, or coronary artery bypass grafting). Early mortality was defined as in-hospital or within 30 days of surgery, if discharged.

**Results:**

The overall early mortality was 10.3% (15/146), and it was significantly different in each Penn Class: 1.5% (1/65) for A, 8.7% (4/46) for B, 22.8% (8/35) for C ( *p* = 0.002). Six patients in Penn Class C had preincision cardiac arrest with cardiopulmonary resuscitation, three surviving. The early mortality differences, however, between the simple (8.3%) and complex (14.0%) operative groups overall and within each Penn Class were not significant. Of the six groups, the lowest mortality was evident in the 41 patients in Penn Class A who had a simple operation, whereas the highest was seen in the 13 Penn Class C patients who underwent a complex operation (0 vs. 23.1%, *p* = 0.001).

**Conclusion:**

In spontaneous acute TAAD, degree of malperfusion on presentation, rather than operative complexity, was the dominant factor in early mortality.

## Introduction

Operative mortality in acute Type A aortic dissection (TAAD) has been reported recently as 17 to 22%, 1 2 3 4 5 with malperfusion syndrome associated with the highest rates. 6 7 8 9 The Penn Classification system, which categorizes levels of malperfusion, has been shown to predict early operative mortality. 10 11 12 Large patient databases of TAAD often have considerable heterogeneity, especially in etiology and chronicity. We sought to apply the Penn Classification to a 6-year series of TAAD patients treated by central repair, specifically those with a spontaneous etiology and acute presentation. Additionally, the impact of operative complexity of the central repair on early mortality was evaluated and its relation to Penn class for prediction of early mortality.

## Materials and Methods

**Fig. 1:**
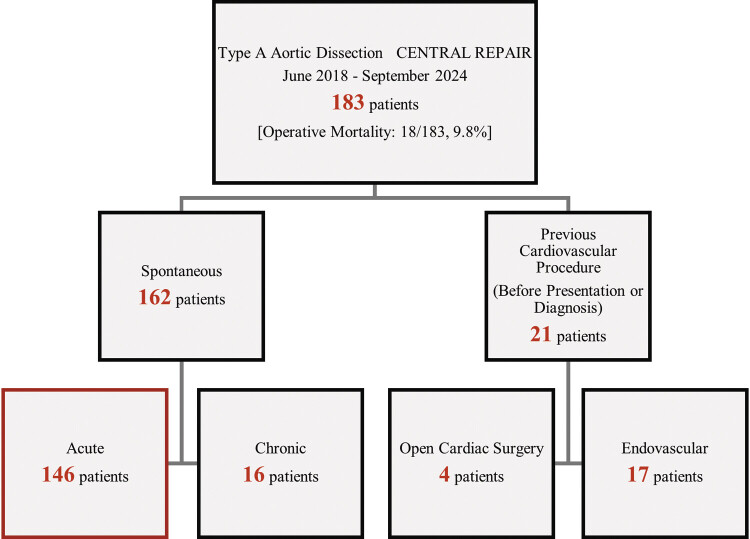
Type A aortic dissection patients distributed by etiology and acuity.

**Fig. 2:**
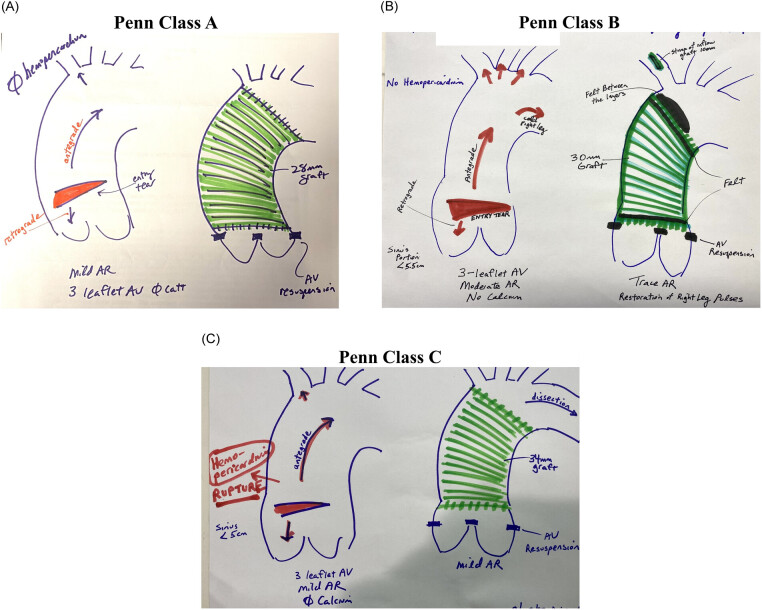
Operative illustrations of a patient in each Penn Class, with similar operations (“simple” by our definition), but the clinical scenarios were worse with advancing Penn Class. ( **A** ) Penn Class A: A 54-year-old woman presented with hypertension and severe chest pain, without malperfusion. Imaging showed a type A aortic dissection with involvement of the ascending aorta only. At operation, the entry tear was near the sinotubular junction, and replacement of the ascending aorta and hemiarch was done, as well as aortic valve resuspension. ( **B** ) Penn Class B: A 47-year-old man presented with chest pain and a cold, painful, and pulseless right lower extremity. Imaging showed a Type A aortic dissection with significant true lumen compression of the right common iliac artery. Following central repair with ascending and hemiarch aortic replacement and aortic valve resuspension for moderate aortic regurgitation (AR), pulses were restored and right leg ischemia was improved. ( **C** ) Penn Class C: A 58-year-old woman with obesity presented with hypotension and severe acidosis, which rapidly progressed to cardiac arrest in the emergency department. Imaging after return of spontaneous circulation demonstrated type A aortic dissection with a pericardial effusion. At operation, aortic rupture with hemopericardium was identified. Ascending and hemiarch replacement with aortic valve resuspension was performed in this patient, who was discharged 11 days later.

This is a single-center, single-surgeon retrospective cohort study of consecutive patients presenting with spontaneous, acute TAAD. This study was reviewed, approved, and due to its retrospective nature, granted a waiver of informed consent by the Baylor Scott and White Research Institute Institutional Review Board (#014-209). A total of 183 patients underwent central repair of TAAD over a 6-year period (June 1, 2018 to September 30, 2024), 146 patients of whom had a spontaneous etiology and acute presentation. Patients who had a previous open cardiac or endovascular aortic procedure were excluded, as well as those with chronic TAAD ( Fig. 1 ). Patient records were reviewed for demographic, procedural, and outcomes data. Preoperative Penn Class was assigned based on history, exam, laboratory, and imaging findings: Class A reflected no malperfusion, Class B regional, and Class C global ( Fig. 2 ). The index operation was identified as simple (ascending aortic with or without hemiarch replacement) or complex (concomitant root replacement, arch replacement, or coronary artery bypass grafting [CABG]).

The technique for central repair of TAAD has been previously described in detail. 13 Briefly, central repairs for TAAD are performed typically with innominate artery cannulation. Replacement of the ascending aorta and hemiarch is the standard operation, defined herein as “simple.” Complete replacement of the aortic root (Bentall procedure) was done for standard indications, typically for Marfan syndrome or aneurysm or coronary ostial destruction. Complete replacement of the arch was done, typically for Marfan syndrome or aneurysm or an entry tear on the greater curve. CABG was added in certain patients with dissection-related myocardial ischemia. Deployment of an endograft in the descending thoracic aorta during circulatory arrest (frozen elephant trunk procedure) was added occasionally, early in the series, or if an entry tear was obvious in the descending thoracic aorta, but this additional endovascular procedure was not a factor in categorizing an operation as simple or complex.

The Society of Thoracic Surgeons definition of early mortality was used: in-hospital or ≤30 days after the index operation. 14 Normality of variables was assessed. Normal continuous variables are presented as a mean (standard deviation), whereas non-normal continuous variables are presented a median (interquartile range). Categorical variables are reported as counts with percentages. Statistical significance for all tests was determined as a *p* -value <0.05. A chi-square test (or Fisher's exact test when low cell counts are present) was used to test for associations in bivariate comparisons. A two-sample *t* -test (or Wilcoxon rank–sum test when appropriate) was used to test for differences in continuous variables between two groups. An analysis of variance (or Kruskal–Wallis test, where appropriate) was used to assess differences in continuous variables between three or more groups.

## Results

Of the 146 patients with spontaneous, acute TAAD treated operatively, 65 were in Penn Class A, 46 in B, and 35 in C. The median age was similar across Penn Classification: 56 [43, 65] years for Penn A, 52 [43, 61] years for Penn B, 58 [52, 69] years Penn C ( Table 1 ). Gender distribution was also similar: 39 (63%) males in A, 30 (77%) males in B, 24 (75%) males C. Medical comorbidities were also similar. Preoperative lactic acid was significantly different across Penn classes: 1.5 mg/dL [1.0, 2.0] for Penn A, 1.9 [1.4, 2.3] for Penn B, and 3.7 [2.6, 5.5] for Penn C ( *p* < 0.001). The presence of moderate to severe medial elastic fiber loss on aortic specimen pathologic analysis was not significantly different between groups. In Penn Class A, the frequency of enlarged sinus of Valsalva (≥5.5 cm in men or ≥5.0 in women) was higher ( *p* = 0.03). In examining the procedures performed across Penn classes, no significant differences in frequency were observed for complete root replacement or complete arch replacement, or the frozen elephant trunk procedure. Overall operative mortality for the cohort was 10.3% (15/146); 1.54% (1/65) for Penn A, 13.0% (6/46) for Penn B, and 22.9% (8/35) for Penn C ( *p* < 0.001). In the six Penn Class C patients who experienced preoperative cardiac arrest, operative mortality was 50% (3/6).

**Table 1:** Clinical variables and early mortality in spontaneous, acute Type A aortic dissection according to Penn class

Penn Class	A	B	C	*p* -Value
*N*	65	46	35	
Age (y)	56 [43, 65]	52 [43, 61]	59 [52, 69]	0.66
Male sex	39 (63%)	30 (75%)	24 (75%)	0.15
Hypertension	51 (82%)	35 (88%)	24 (75%)	0.36
Diabetes mellitus	5 (8.1%)	5 (13%)	3 (9.4%)	0.70
End-stage renal disease	2 (3.2%)	1 (2.5%)	1 (3.1%)	0.93
Current smoking	30 (48%)	22 (55%)	21 (66%)	0.21
Preop lactic acid (mg/dL)	1.5 [1.0, 2.0]	1.9 [1.4, 2.3]	3.7 [2.6, 5.5]	<0.001
3–4+ aortic medial EFL	8 (13%)	1 (2.5%)	3 (9.4%)	0.20
Enlarged SoV	14 (23%)	3 (7.5%)	2 (6.3%)	0.03
Entry tear location				0.35
Zone 0A	44 (71%)	28 (70%)	18 (56%)	
> Zone 0A	18 (29%)	12 (30%)	14 (44%)	
Aortic valve replacement	0	0	1 (2.9%)	0.97
Root replacement	13 (21%)	5 (13%)	6 (19%)	0.51
Arch replacement	8 (12%)	2 (4.3%)	4 (11%)	0.76
Frozen elephant trunk	11 (17%)	14 (30%)	14 (40%)	0.14
CABG	3 (4.6%)	6 (13%)	2 (5.7%)	0.74
Operative mortality	1 (1.5%)	6 (13%)	8 (23%)	<0.001

Abbreviations: CABG, coronary artery bypass grafting; EFL, elastic fiber loss; SoV, sinus of Valsalva.

Stratified by operative complexity, early mortality for simple operations was 0% (0/41) for Penn A, 9.1% (3/33) for Penn B, and 22.7% (5/22) for Penn C ( Table 2 ; *p* = 0.007). For complex operations, early mortality was 4.2% (1/24) for Penn A, 23.1% (3/13) for Penn B, and 14.0% (7/50) for Penn C ( *p* = 0.164).

**Table 2:** Distribution of patients and early mortality in Type A aortic dissection, stratified by operative complexity and Penn Class

Operation	Penn A	Penn B	Penn C	Total	*p*
Simple a	41 (0%)	33 (9.1%)	22 (22.7%)	96 (8.3%)	0.007
Complex b	24 (4.2%)	13 (23.1%)	13 (23.1%)	50 (14.0%)	0.164
Total	65 (1.5%)	46 (8.7%)	35 (22.8%)	146 (10.3%)	0.002
*p* -Value	0.193	0.213	0.982	0.288	

a ascending aortic with or without hemiarch replacement.

b Concomitant root replacement, arch replacement, or coronary artery bypass grafting.

## Discussion

Until the Penn Classification system of aortic dissection was reported in 2009, most systems were based simply on involvement of aorta. The classification by ischemia, or malperfusion, was new. This study supports the view that stratifying patients by the level of ischemia on presentation is a superb predictor of early mortality. Minimal malperfusion is associated with minimal mortality, and maximal malperfusion is associated with maximal mortality. The operation itself, provided it is a standard procedure done in a timely way, appears to be less important than the clinical presentation, in predicting outcome. A TAAD patient without malperfusion has a similar operative risk as a coronary bypass, at least in this series.

Malperfusion has always been associated with poor outcomes in TAAD. In a multicenter analysis, Zindovic et al. noted mortality rates in TAAD of 29% in patients with malperfusion syndrome compared with 12% in patients without. 15 Our findings lend support to previous studies demonstrating a similar trend of increased mortality associated with advanced Penn Class. 11 It may be a consideration to create a “Penn Class D” for patients who have a preincision cardiac arrest. In our group of six patients, we observed a significantly higher early mortality, within the Penn C group: 50% (3/6) versus 19% (5/26). Importantly, the Penn Classification system makes no stratification by preoperative cardiac arrest events.

The early mortality differences between the simple (8.3%) and complex (14.0%) operative groups overall (and within each Penn Class) were not significant. The large apparent difference in mortality between the two groups with nonsignificance ( *p* = 0.288) on statistical analysis suggests that our analysis was underpowered to detect such differences, which is a limitation of this study. Perhaps in a larger series, an early mortality difference may appear between simple and complex operations. Of the six groups, the lowest mortality was evident in the 41 patients in Penn Class A who had a simple operation, whereas the highest was seen in the 13 Penn Class C patients who underwent a complex operation (0 vs. 23.1%, *p* = 0.001).

## Conclusion

In summary, the level of ischemia (none, regional, or global) on presentation in TAAD remains a superb predictor of early operative outcome, perhaps more so than the complexity of the operation. Penn Class A correlates with minimal early mortality and Penn Class C, maximal. To address the higher early mortality in Penn Classes B and C, delaying central repair after specific treatment of regional malperfusion in some cases may be beneficial.
